# Revisiting pulmonary fibrosis: inflammatory dynamics of the lipofibroblast-to-inflammatory lipofibroblast-to-activated myofibroblast reversible switch

**DOI:** 10.3389/fimmu.2025.1609509

**Published:** 2025-06-18

**Authors:** Georgios-Dimitrios Panagiotidis, Esmeralda Vasquez-Pacheco, Xuran Chu, Werner Seeger, Elie El Agha, Saverio Bellusci, Arun Lingampally

**Affiliations:** ^1^ Department of Respiratory and Critical Care Medicine, Quzhou People’s Hospital, The Quzhou Affiliated Hospital of Wenzhou Medical University, Quzhou, Zhejiang, China; ^2^ Department of Medicine V, Internal Medicine, Infectious Diseases and Infection Control, Universities of Giessen and Marburg Lung Center (UGMLC), German Center for Lung Research (DZL), Justus-Liebig University Giessen (JLU), Giessen, Germany; ^3^ Department of Medicine II, Internal Medicine, Pulmonary and Critical Care, Universities of Giessen and Marburg Lung Center (UGMLC), German Center for Lung Research (DZL), Justus-Liebig University Giessen, Giessen, Germany; ^4^ Cardio-Pulmonary Institute (CPI), Giessen, Germany; ^5^ Institute for Lung Health (ILH), Giessen, Germany; ^6^ Oujiang Laboratory (Zhejiang Lab for Regenerative Medicine, Vision and Brain Health), School of Pharmaceutical Science, Wenzhou Medical University, Wenzhou, Zhejiang, China

**Keywords:** idiopathic pulmonary fibrosis, lipofibroblast, inflammatory lipofibroblast, activated myofibroblast, TGF-β, IL-17A, inflammation, virus infection

## Abstract

Idiopathic pulmonary fibrosis (IPF) is a chronic, progressive interstitial lung disease characterized by excessive extracellular matrix (ECM) deposition and irreversible lung damage. A key driver of disease progression is the phenotypic shift of lipofibroblasts (LIFs) into activated myofibroblasts (aMYFs), triggered by sustained epithelial injury, caused by inflammation, oxidative stress, viral infections (e.g., influenza, SARS-CoV-2), and metabolic dysfunction. Emerging evidence demonstrates that this transition is reversible, with pharmacological agents that promote aMYF-to-LIF reprogramming contributing to fibrosis resolution. The identification of inflammatory lipofibroblasts (iLIFs) highlights the importance of inflammation in fibrosis progression. Inflammation, mediated by IL-1β, IL-17A, and TGF- β, sustain aMYF activation, while immune cells shape fibrosis formation. This review combines current insights on the cellular and molecular pathways controlling fibroblast differentiation, highlighting key metabolic, immunologic, and oxidative stress-modulating targets for therapeutic intervention. Understanding and manipulating the LIF-iLIF-aMYF axis offers a promising strategy for reversing fibrosis and restoring pulmonary homeostasis in IPF.

## Introduction

Fibrosis is a progressive and chronic pathological process (1) that can lead to permanent tissue damage, organ dysfunction, and failure, as seen in kidney and liver diseases and idiopathic pulmonary fibrosis (IPF) (2). IPF is a severe, end-stage lung disease with high mortality rates (3) and current treatments-nintedanib and pirfenidone, approved by the United States Food and Drug Administration (FDA) in 2014, are limited to slowing down disease progression (4). In IPF, the alveolar architecture-essential for homeostasis and gas exchange, is disrupted, leading to impaired lung function (1). This is caused by the excessive deposition of extracellular matrix (ECM) components, such as collagens and glycoproteins (3, 5), which, under normal conditions, play a critical role in tissue repair (6).

Fibrogenesis is a complex process involving multiple pathophysiological mechanisms, cell types, and signaling pathways (3). Initially, IPF was thought to result from chronic inflammation (1). However, the failure of immunosuppressive therapies to improve fibrosis outcomes led to a reevaluation of this hypothesis (7). Recent evidence suggests that IPF arises from chronic epithelial injury triggered by factors such as persistent oxidative stress (8), inflammation (9), viral infections (10), metabolic dysregulation (3, 11) aging (12) and genetic background (13, 14).

**Figure 1:**
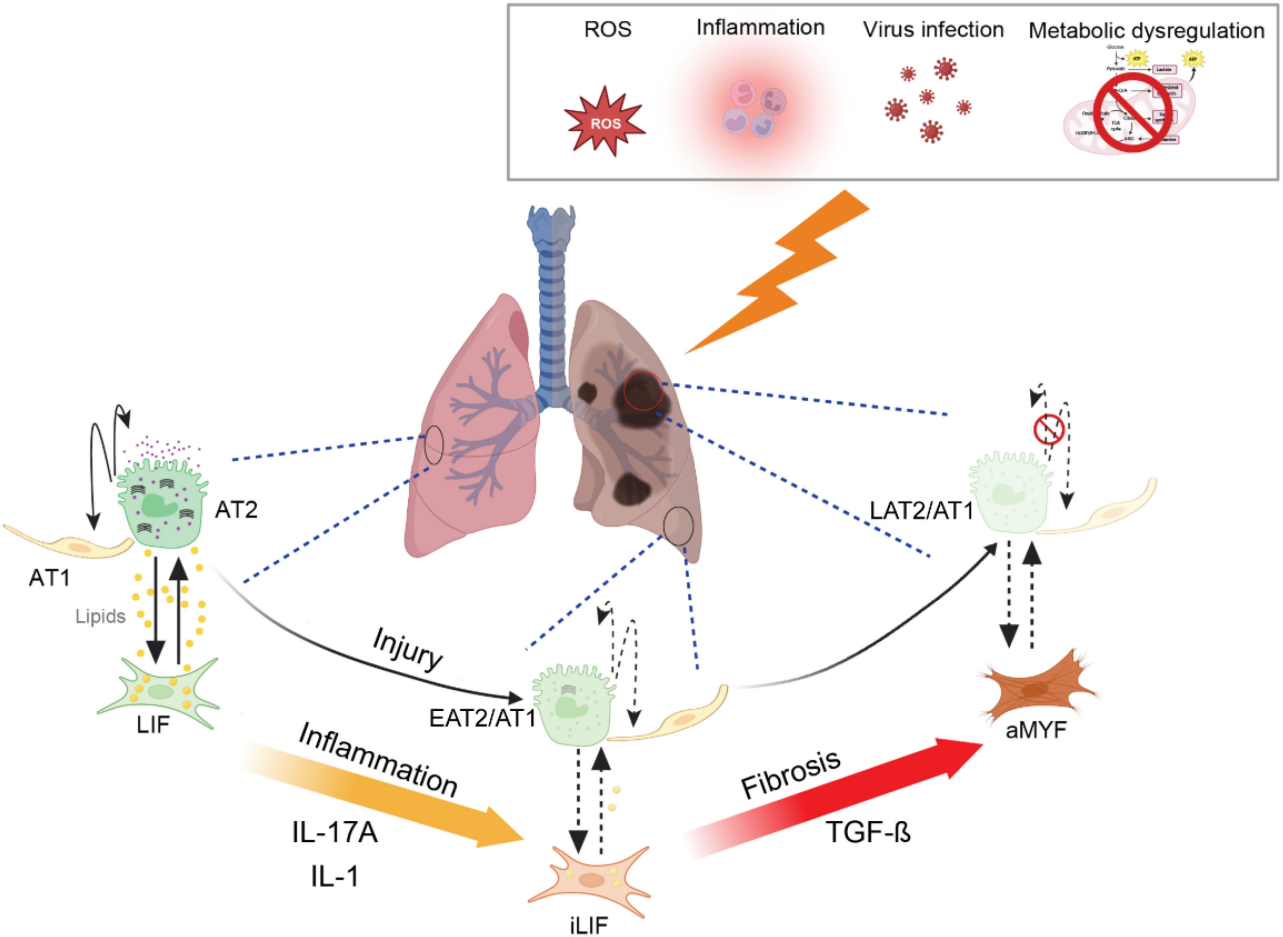
Schematic representation of lung fibrosis progression and its cellular and metabolic mechanisms. In healthy lung, alveolar epithelial cells (AT1 and AT2) maintain normal gas exchange, with lipofibroblasts (LIF) providing support to the alveolar niche. Under persistent oxidative stress (ROS), inflammation, virus infection and metabolic dysregulation, LIF transition into intermediate inflammatory lipofibroblasts (iLIFs) (18), leading to impaired alveolar epithelial stem cell niche. Persistent injury, inflammation and activation of myofibroblasts (aMYF) result in fibrosis, disrupting alveolar architecture and leading to loss of gas exchange. The progressive pathological changes ultimately contribute to impaired lung function. EAT2/1: early intermediate AT2/AT1, LAT2/AT1: late intermediate AT2/AT1.

Following epithelial injury, the wound healing process is initiated, involving the recruitment of fibroblasts and their differentiation into myofibroblasts, which deposit ECM components to facilitate wound closure (15, 16). This process is regulated by Transforming growth factor-β (TGF-β). TGF-β plays a central role in fibrosis development, and its levels correlate with disease severity (17). TGF-β not only promotes fibroblast-to-myofibroblast differentiation but also exacerbates inflammation and oxidative stress, creating a self-perpetuating cycle of injury and fibrosis (**Figure 1**) (3, 9).

Emerging research highlights the critical role of lipofibroblasts in fibrosis resolution. Lipofibroblasts (LIFs) are specialized fibroblasts within the alveolar niche that play a crucial role in lung development, homeostasis, and repair. They are characterized by their lipid storage capabilities and close association with alveolar epithelial type 2 (AT2) cells (19–22). LIFs are in close proximity to AT2 cells, and play a key role in regulation of inflammation, alveolar development and lung homeostasis (21, 23). LIFs transdifferentiate into activated myofibroblasts (aMYFs) during fibrosis. Lingampally et al., 2025 (12) demonstrate that aging leads to a phenotypic shift in alveolar fibroblasts from lipofibroblasts (LIFs), which support alveolar type 2 (AT2) stem cells, toward aMYFs. Using lineage tracing and scRNA-seq, the study shows that aged mice exhibit an increased proportion of aMYFs and reduced expression of LIF markers during fibrosis, with delayed resolution compared to young mice. Importantly, alveolosphere assays revealed that alveolar fibroblasts isolated from aged mice lack the ability to support AT2 cells, resulting in a complete loss of alveolosphere formation *in vitro*. This indicates that the impairment lies within the aged mesenchymal niche, not the epithelial stem cells. These findings provide strong evidence that the aging lung mesenchyme negatively impacts AT2 cell renewal by losing supportive LIF identity in favor of a profibrotic aMYF phenotype (12). This phenotypic switch contributes to the development and progression of lung fibrosis. Conversely, in the mouse bleomycin-model, the reversion from myofibroblasts back to lipofibroblasts has been observed during fibrosis resolution offering a potential therapeutic target (12, 24–28).

Epithelial injury is widely recognized as a central initiating event in the development of IPF. Among epithelial cell populations, AT2 cells play a critical role in maintaining alveolar homeostasis through their ability to self-renew and transdifferentiate into alveolar type 1 (AT1) cells (22). In IPF, this regenerative capacity is impaired, leading to the accumulation of dysfunctional or ‘diseased’ AT2 cells. These cells often adopt an intermediate phenotype and show defective differentiation (29), thereby contributing to pathological remodeling. Moreover, AT2 have been shown to secrete pro-fibrotic mediators that promote fibroblast activation and extracellular matrix deposition (29, 30).

Future research focusing on developing pharmaceuticals targeting the underlying causes of IPF and exploring the reversible transition between myofibroblasts towards lipofibroblasts to improve patient outcomes is promising. In this review, we provide new insights into the mechanisms driving IPF and emphasize the importance of the activated myofibroblast-to-lipofibroblast switch in fibrosis resolution.

## Pathophysiological conditions leading to fibrosis

### Virus infection

Chronic and recurring viral infections can contribute to the development of IPF. This occurs through two primary mechanisms: direct damage caused by the virus and the immune response triggered as a host defense. Viral infections directly injure the distal lung epithelium, and when this damage is persistent or repeated, it activates fibroblast and disrupts normal wound healing, leading to fibrosis. The precise identity of the activated fibroblast upon virus infection is still unclear. However, as their activation is associated with damage to the AT2 cells, it is likely that these activated fibroblasts are indeed lipofibroblasts. Simultaneously, the host immune response to the virus initiates inflammation. Inflammatory cells infiltrate the lung epithelium, releasing pro-fibrotic and pro-inflammatory factors such as TGF-β and Interleukins (e.g., IL-1, IL-17), which perpetuate myofibroblast activation (17, 31). This section examines the impact of common and recurrent viral infections, specifically those caused by influenza viruses and SARS-CoV-2, on the pathogenesis and progression of IPF.

#### Influenza virus

Influenza A (H1N1) virus is a major respiratory pathogen responsible for seasonal infections, causing an estimated 290,000–650,000 deaths worldwide annually (WHO Influenza (Seasonal), 2023). The 1918 and 2009 H1N1 pandemics were particularly devastating, resulting in approximately 40 million and 151,700–575,400 deaths, respectively (32, 33). H1N1 infection is a known causal factor of pulmonary fibrosis (34), with evidence suggesting that fibroblast activation (17, 31), as part of the repair process following virus-induced inflammation, contributes to fibrosis. Conversely, single-cell transcriptomic studies indicate that alveolar myofibroblast (AMF)-like cells emerge during alveolar regeneration after influenza-virus-induced lung injury, resembling fibrosis-associated myofibroblasts (FAMs) (35). Bioinformatic analysis of the AMF-like cells indicated that they contribute to the alveolar fibroblast pool which contains the LIFs (25, 35). Dysregulation of AMF-like cells has been linked to failed alveolar regeneration and non-resolving fibrosis, particularly in cases of severe acute respiratory distress syndrome (ARDS).

H1N1 infection has been shown to upregulate TGF-β expression in alveolar epithelial cells (31) and primary tracheal epithelial cells (36), potentially exacerbating fibrotic responses. Additionally, studies demonstrate that AMF-like cells in influenza-induced lung injury upregulate fibrotic markers such as Alpha smooth muscle actin (α-SMA/ACTA2) and Collagen triple helix repeat containing 1 (CTHRC1), further suggesting that their over-activation can lead to fibrosis development (35).

Avian influenza viruses, such as H7N9 and H5N1, also pose a significant threat to humans, with certain strains being highly pathogenic and fatal (37–39). Avian influenza has been implicated in the development of pulmonary fibrosis. For instance, patients recovering from severe H7N9-induced pneumonia, developed fibrosis (40). In a fatal case report, H7N9 infection led to rapid progression of pulmonary fibrosis (41). Disease severity is linked to elevated plasma angiotensin II (Ang-II) levels, which are induced by H7N9 infection and associated with increased mortality (42). Ang-II, a component of the renin-angiotensin system (RAS), promotes fibrosis by upregulating TGF-β, Nuclear factor kappa B (NF-κB) expression (43) and enhancing reactive oxygen species (ROS) production in lung fibroblasts, thereby stimulating their migration and collagen synthesis (44). Conversely, angiotensin-converting enzyme 2 (ACE2) counteracts fibrosis by mitigating the effects of Ang-II. In another case report, fatal H5N1 infection was associated with interstitial fibrosis (45). Mouse models support these findings, where H5N1 infection triggered an inflammatory response, increased ECM deposition, and ultimately led to fibrosis (46, 47). ACE2 also plays a protective role in H5N1-induced fibrosis, as genetic ablation of *Ace2* exacerbated lung fibrosis, while recombinant ACE2 reduced viral replication and attenuated fibrosis (48).

#### SARS-CoV-2

Severe acute respiratory syndrome coronavirus 2 (SARS-CoV-2), the virus responsible for the COVID-19 pandemic, has caused over 800 million infections and 7 million deaths worldwide as of November 2024 (WHO COVID-19 Dashboard, n.d.). SARS-CoV-2 infection is associated with the development of lung fibrosis, as evidenced by biopsies from COVID-19 patients (49, 50).

The origin of the activated MYF is still unclear. However, studies by our group and others indicated that the alveolar fibroblast, which includes the LIFs, massively contributed to the activated myofibrobasts (12, 18, 26, 51–53).

Studies have shown that survivors of acute COVID-19 pneumonia exhibit fibrotic changes in the lungs, detectable 6 months after discharge (54). SARS-CoV-2-induced pulmonary fibrosis is linked to an excessive immune response, immune cell infiltration, and fibroblast proliferation (55).

Lung fibrosis in COVID-19 is thought to result from persistent injury caused by the virus and excessive host inflammatory response (10). SARS-CoV-2 binds to the ACE2 receptor during viral entry. Several studies have demonstrated the connection between SARS-CoV-2 infection, ACE2, and TGF-β in fibrosis development. SARS-CoV-2 downregulates ACE2, disrupting the angiotensin II (Ang II), which promotes inflammation and fibrosis (56). The infection also increases the expression of Tumor necrosis factor-alpha (TNF-α) (57), a cytokine associated with fibrosis (58), and inflammatory interleukins IL-1β, IL-6, IL-8, IL-18 (59) IL-2R and IL-10 (60).

Bioinformatic analyses have revealed that SARS-CoV-2 binding to ACE2 upregulates fibrotic mRNA markers, including *TGF-beta*, Vascular endothelial growth factor A (*VEGFA*), Connective tissue growth factor (*CTGF*), and Fibronectin 1 (*FN1*), all identified as ACE2-interacting molecules (61).

Collectively, these findings underscore the significant contribution of viral infections to the initiation and progression of fibrosis, highlighting the specific molecular pathways involved in this pathogenic process.

### Oxidative stress

Oxidative stress arises from an imbalance between the production of reactive oxygen species (ROS) and the cellular antioxidant defense mechanisms (62). ROS are primarily generated as byproducts of mitochondrial electron transport chain dysfunction (63). In IPF, ROS and TGF-β are closely interconnected, forming a vicious cycle of positive feedback loop. ROS can directly activate TGF-β1 and enhances ROS production, which in turn upregulates TGF-β expression, exacerbating fibrotic processes (64, 65).

NADPH oxidase 4 (NOX4), a key ROS-generating enzyme, plays a significant role in fibrosis. TGF-β induces NOX4 expression during fibrotic formation, and studies have demonstrated that genetic or pharmacological inhibition of *Nox4* attenuates fibrosis progression in murine models (66). The critical role of ROS in fibrosis is further supported by studies showing the protective effects of antioxidants. In bleomycin-induced fibrosis models, antioxidants such as superoxide dismutase (SOD), catalase, porphyrin MnTBAP, N-acetylcysteine, and lazaroids have been shown to mitigate lung damage in mice and rats (67, 68).

ROS promote inflammation through multiple interconnected molecular pathways, primarily by disrupting cellular redox balance, activating transcription factors, and enhancing the production of proinflammatory cytokines and chemokines (8). One of the primary way ROS drive inflammation is through the activation of the NF-κB signaling pathway. Under normal conditions, NF-κB is sequestered in the cytoplasm by its inhibitor, IκBα. ROS cause oxidative modifications that lead to the degradation of IκBα via the IκB kinase (IKK) complex, releasing NF-κB, which then translocates to the nucleus and induces the expression of proinflammatory genes such as *TNF-α*, *IL-6*, and *IL-1β​* (69, 70). Thus, ROS act as key mediators of inflammation by activating transcription factors (NF-κB), signaling pathways (TGF-β), all of which contribute to chronic lung inflammation and fibrosis.

**Figure 2:**
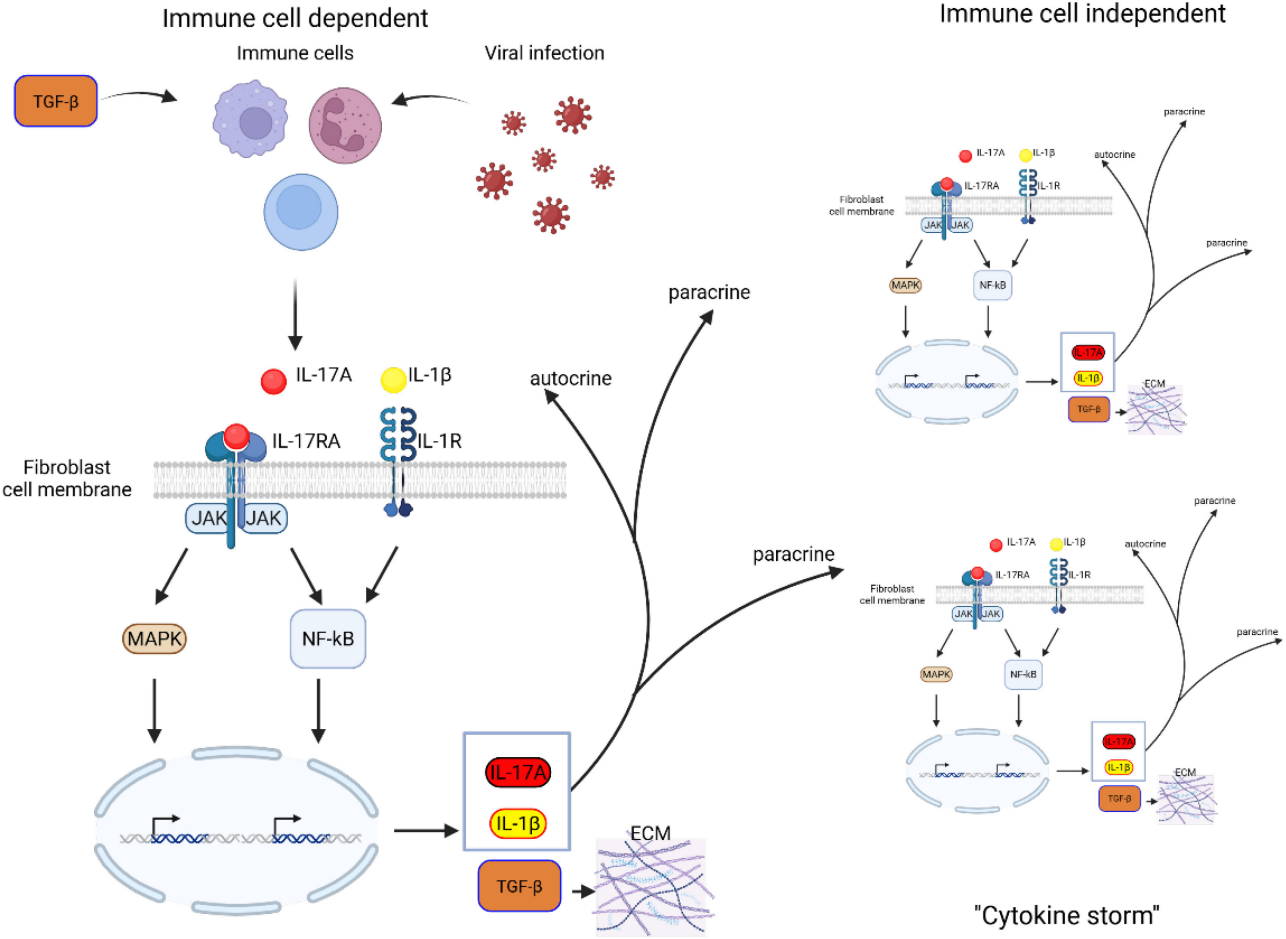
Schematic representation of IL-17A-TGF-β positive feedback loop in fibroblasts. TGF-β stimulation of immune cells as well as inflammatory response to virus infection induces IL-17A. IL-17A binds to its receptor IL-17RA, activating downstream signaling pathways, including MAPK and NF-κB, which drive gene transcription. This leads to the production of IL-17A, IL-1β, and TGF-β, amplifying inflammatory responses. IL-17A and IL-1β act in a paracrine and autocrine reinforcing the inflammatory loop. Increased TGF-β expression promotes ECM deposition, contributing to fibrosis and tissue remodeling. Persistent activation of these pathways may lead to excessive fibrotic responses, disrupting normal tissue architecture and function.

**Figure 2** shows the consequences of such activation in the fibroblasts in general, not only in the context of ROS. In the context of the lung, we propose that these fibroblasts identity with Lipofibroblasts and that their activation leads not only to their transition towards the activated MYF but also to the production of interleukines, leading to the detrimental “cytokine storm”.

Aldehyde dehydrogenase 2 (ALDH2), a major antioxidant enzyme, also plays a pivotal role in fibrosis progression. In a study by Tan et al., 2021 (71), ALDH2 transcription and protein levels were significantly reduced in fibroblasts from both bleomycin-induced IPF mouse models and human IPF lungs. Genetic enhancement of *ALDH2* using CRISPR in human lung fibroblasts resulted in decreased expression of profibrotic genes (*ACTA2, COL1A1*), reduced ECM deposition, and diminished fibroblast contractility, even in the presence of TGF-β. These findings underscore the critical role of ALDH2 in modulating fibrotic processes (71).

Overall, these findings highlight the central role of oxidative stress in the pathogenesis of IPF, where ROS-mediated signaling pathways, inflammation, and fibrotic processes create a self-perpetuating cycle that exacerbates disease progression.

### Metabolic dysregulation

Lipid and carbohydrate metabolism play significant roles in the progression and resolution of IPF. Factors such as TGF-β, which are critical in IPF development, also exert substantial effects on cellular metabolism (11).

Emerging evidence highlights the profound impact of TGF-β on cellular metabolism. In human fibroblasts, TGF-β upregulates glycolytic genes, glucose transporters (72) glutamine synthase (73) and overall glycolysis (74). This is further supported by findings that inhibition of glycolysis blocks TGF-β-induced α-SMA expression and myofibroblast differentiation (75). Given these observations and the beneficial role of lipofibroblasts in IPF resolution, it is evident that both carbohydrate metabolism and lipid metabolism are crucial in IPF pathogenesis.

Glycolysis, a central metabolic pathway, generates energy and produces amino acids, nucleotides, and lipids. Under aerobic conditions, glycolysis metabolizes one glucose molecule into 32 ATP molecules, whereas anaerobic conditions yield only 2 ATP molecules (76). Numerous studies have linked glycolysis to IPF (77–82). For instance, TGF-β induces glucose uptake during fibrogenesis by upregulating Glucose transporter 1 (GLUT1), a downstream effect of TGF-β signaling (77). GLUT1 is also associated with fibroproliferation in aged human lungs and bleomycin-induced IPF models (78). Additionally, glycolytic enzymes such as hexokinase and lactate dehydrogenase (LDH) are elevated in IPF patients (79). In the study of Yin et al., 2019 (80) hexokinase is upregulated by TGF-β, and inhibition of hexokinase show anti-fibrotic effect. Under anaerobic conditions, glucose is metabolized into lactate, which has profibrotic properties (79). Inhibition of LDH, the enzyme responsible for lactate production, suppresses TGF-β-induced myofibroblast differentiation. Metabolic analyses of fibroblasts and myofibroblasts from IPF patients reveal increased glycolysis, a phenomenon replicated in TGF-β-treated fibroblasts (81, 82). This glycolytic shift is accompanied by elevated myofibroblast markers like ACTA2, which are reduced upon glycolysis inhibition (82).

On the other hand, lipid metabolism is essential for lung function, as the lungs are lipid-rich organs (83). Lipids play structural roles, such as the production of lipid-derived surfactants by alveolar epithelial type 2 cells, which maintain alveolar structure by reducing surface tension (84, 85). Lipids are also involved in ECM composition, cellular communication, signaling, and inflammation (84, 85). Lipid metabolism produces over 100 ATP molecules per cycle while also generating substantial amounts of ROS (86, 87). Studies have demonstrated dysregulation of lipid metabolism in IPF (88–90). Transcriptomic analyses reveal downregulation of genes involved in lipid metabolism, synthesis, and transport in human AT2s and epithelial cells (88). Conversely, alveolar macrophages from IPF patients exhibit enrichment of lipid metabolism genes, leading to extracellular lipid accumulation (89, 90) This lipid accumulation enhances CD36 expression in macrophages. Genetic ablation of *Cd36*, a fatty acid receptor, reduces profibrotic cytokine production and alleviates fibrosis in bleomycin-induced models (91).

Lipid metabolism also influences fibroblast fate and fibrosis progression. TGF-β treatment of human lung fibroblasts induces a myofibroblast phenotype characterized by increased collagen production and suppression of Peroxisome proliferator-activated receptor gamma (PPAR-γ), a lipofibroblast marker (25). Treatment with rosiglitazone, a PPAR-γ agonist, reverses this lipo-to-myogenic differentiation. Furthermore, metformin, a multifunctional metabolic modulator, reverses TGF-β-mediated myofibroblast differentiation, enhances fibroblast functionality in organoid formation (26) and promotes fibrosis resolution in bleomycin-induced fibrosis models (28, 92). Additionally, metformin was employed in *in vivo* models of IPF, resulting in improved survival and lung function, and, importantly, the restoration of AT2 cell mitochondrial respiration and a reduction in cytokine production, collectively contributing to the mitigation of epithelial injury (93).

While PPAR-γ has been recognized for its anti-fibrotic properties, the role of PPAR-β/δ remains less clear. The study of Boateng et al., 2024 (94) investigates the interplay between Peroxisome proliferator-activated receptors (PPAR-β/δ and PPAR-γ) in the pathogenesis of idiopathic pulmonary fibrosis. The study suggests that both receptors modulate fibroblast activity, ECM deposition, and inflammatory signaling, ultimately affecting towards lung fibrosis resolution. Using lung fibroblasts from healthy and IPF patients this study demonstrates that PPAR-β/δ negatively modulates fibroblast-to-myofibroblast differentiation, a critical step in IPF progression, potentially through interaction with TGF-β signaling. PPAR-β/δ may negatively regulate genes involved in cytoskeletal remodeling, such as *Acta2* and *Fn1*, influencing fibroblast contractility collagen synthesis and ECM turnover by interacting with MMPs (matrix metalloproteinases) and TIMPs (tissue inhibitors of metalloproteinases), balancing fibrosis and ECM degradation.

These findings underscore the critical roles of lipid and carbohydrate metabolism in IPF pathogenesis, highlighting their influence on fibroblast differentiation, ECM remodeling, and fibrotic progression, with potential therapeutic implications for metabolic modulation in fibrosis treatment. Given its regulatory role in fibrosis, targeting PPAR-β/δ with agonists could provide therapeutic benefits. Further research is required to determine the precise molecular interactions of PPAR-β/δ in IPF and assess its potential as a therapeutic target and connecting it with lipofibroblast-to-activated myofibroblast- reversible switch.

## Lipofibroblast-to-activated myofibroblast reversible switch

Fibroblasts are a ubiquitous and fundamental cell type within connective tissues, known for their role in ECM production and tissue homeostasis (95). The term “fibroblast” has been present in the scientific literature for over a century and was initially described as a connective tissue cell responsible for ECM synthesis (96). Historically, fibroblasts were believed to be derived from fibrocytes, with an assumed dynamic transition between these cell states depending on tissue damage and repair (97). Morphologically, fibroblasts exhibit a spindle- or stellate-shaped structure and display substantial heterogeneity, participating in various physiological and pathological processes, including development and fibrosis (98).

The classical view of fibroblast plasticity has evolved with recent evidence supporting a reversible transition between lipofibroblasts (LIFs) and activated Myofibroblasts (aMYFs) during fibrosis and its resolution (25, 26, 52, 99). Lipofibroblasts are characterized by intracellular lipid droplets and adipocyte-like properties, marked by the expression of Perilipin 2 (PLIN2). These cells play a critical role in supporting AT2 cells by promoting surfactant production (19–21). Conversely, myofibroblasts are defined by their expression of α-SMA/ACTA2 and are responsible for ECM deposition, playing essential roles in wound healing and pathological fibrosis. Increasing evidence suggests that myofibroblasts originate from lipofibroblasts and that pharmacological reprogramming to promote lipofibroblast identity may facilitate fibrosis resolution and present therapeutic potential (25, 26, 52).

El Agha et al., 2017 (25) employed lineage tracing to investigate the transition between lipofibroblasts and activated myofibroblasts during fibrosis. Their study demonstrated that lipofibroblasts serve as progenitors of activated myofibroblasts during fibrosis progression. Furthermore, activated myofibroblasts could revert to a lipofibroblast-like phenotype during resolution, and pharmacological induction of a lipogenic profile counteracted the myogenic differentiation induced by TGF-β, suggesting a potential intervention for IPF. Vásquez-Pacheco et al., 2024 (26) corroborated these findings using the human lung embryonic fibroblast cell line WI-38. Their transcriptomic analysis reinforced the lineage relationship between lipofibroblasts and myofibroblasts. Additionally, organoid formation assays demonstrated that metformin, an antidiabetic drug, induced a lipogenic shift in myofibroblasts, enhancing alveolosphere formation (26, 28). Lingampally et al., 2025 (52) further supported these findings using a bleomycin-induced IPF model in mice and human IPF lungs (52). Through lineage tracing, the authors proposed that activated myofibroblasts, marked by *Cthrc1*, originate from *Acta2*-negative, LIF^high^ alveolar fibroblasts. These activated myofibroblasts exhibit a fibrotic signature and progressively differentiate from *Cthrc1* ^low^ LIF^high^ to *Cthrc1* ^high^ cells. During fibrosis resolution, the reverse transition was observed, wherein *Cthrc1* ^high^ cells reverted to *Cthrc1* ^low^ LIF^high^ and eventually to Acta2-negative LIF^high^ fibroblasts. Notably, *Cthrc1*+ activated myofibroblasts have been implicated as key drivers of fibrosis development (100).

Fibroblast heterogeneity in the lung has been increasingly recognized as a key factor in both homeostasis and fibrosis. Recent studies have identified a transitional inflammatory fibroblast population that emerges early in the fibrotic response, bridging the gap between homeostatic states. In the study by Tsukui et al., 2024 (18), the authors utilized a *Scube2^CreERT2^* mouse model to specifically label alveolar fibroblasts.

*Scube2* is a marker of a specialized alveolar fibroblast population that supports epithelial progenitors and shares transcriptional features with lipofibroblasts, including genes involved in lipid metabolism and surfactant regulation. *Scube2* ^+^ fibroblasts localize near AT2 cells, express lipid-associated genes such as *Plin2* and *Ppar-g*, and transition toward a more inflammatory or activated myofibroblast-like state under fibrotic conditions, supporting our hypothesis of a lipo-inflammatory-myofibroblast (LIF) trajectory and reinforcing the functional relevance of *Scube2* ^+^ lipofibroblast-like cells in lung homeostasis and repair. Therefore, we propose that the lipofibroblasts represent a functional phenotype, while *Scube2* ^+^ fibroblasts are defined by a transcriptional and spatial signature. *Scube2* ^+^ alveolar fibroblasts constitute a lipofibroblast-enriched subset, rather than being entirely synonymous (18, 53, 101).

These fibroblasts were found to express characteristic markers such as Nephronectin (*Npnt*), Platelet-derived growth factor receptor alpha (*Pdgfra*), and *Cd34*. Beyond their homeostatic role, alveolar fibroblasts serve as progenitors for an intermediate inflammatory fibroblast population that emerges in response to tissue injury and inflammation. These inflammatory fibroblasts are characterized by the expression of chemokines such as (*Cxcl12)*, Serum amyloid A3 (*Saa3*), and Lipocalin 2 (*Lcn2*). Their induction is driven by pro-inflammatory cytokines, including Interleukin-1β (IL-1β) and Tumor necrosis factor (TNF). This is also supported by the findings of (12) where interleukin-1 (IL-1) and interleukin-17A (IL-17A) signaling are upregulated during fibrinogenesis more prominently in old, aged mice using a murine fibrotic model. This transient inflammatory state is subsequently regulated by TGF-β, which suppresses inflammatory gene expression and promotes differentiation into activated myofibroblasts. This dynamic fibroblast plasticity highlights a complex regulatory network that governs the balance between repair and pathological fibrosis. The differentiation of inflammatory fibroblasts into fibrotic myofibroblasts contributes to fibrosis, emphasizing the need for targeted interventions that can modulate fibroblast fate decisions to mitigate progressive fibrotic remodeling. Further characterization of inflammatory fibroblasts has revealed that they possess a unique gene expression profile that distinguishes them from other fibroblast populations. They exhibit upregulation of interferon-responsive genes and stress-associated markers, suggesting a critical role in the early immune response to lung injury. These cells are found in close proximity to immune cell infiltrates, particularly monocytes and γδ T cells, which contribute to the inflammatory microenvironment (18).

The balance between inflammation and fibrosis appears to be a tightly regulated process, where inflammatory fibroblasts serve as a key intermediary linking tissue injury, immune activation, and fibrotic remodeling (18).

Collectively, these findings highlight the crucial role of the dynamic transition between lipofibroblasts, inflammatory fibroblasts, and activated myofibroblasts in the progression and resolution of fibrosis. Modulating this cellular plasticity towards lipogenic and anti-inflammatory interventions presents a potential therapeutic strategy for IPF.

## Inflammatory response

The inflammatory response is closely linked to wound healing and fibrosis. Numerous studies suggest that IPF involves a shift towards an inflammatory response (3). Proinflammatory cytokines, particularly IL-1β and IL-17A, play a central role in this process (12, 18, 27). Interleukins (ILs) are cytokines that regulate maturation, migration, adhesion, and inflammatory responses through both paracrine and autocrine mechanisms (102). IL-1β is produced in response to cytokine stimulation (103) and was shown to promote fibrosis (104–106) in an IL-17A dependent manner (105, 106).

The study by Wilson et al., 2010 (106) highlighted the interplay between IL-1β, IL-17A, and TGF-β. Using IL-1β and bleomycin-induced fibrotic models, the authors found that progression of fibrosis is dependent on IL-17A, whose expression is regulated by TGF-β, establishing an IL-1β–IL-17A–TGF-β axis. Additionally, IL-17A expression was shown to induce TGF-β production. IL-17A also enhances TGF-β stability, increasing fibroblast sensitivity to TGF-β and ECM production (107). The profibrotic role of IL-17A is supported by evidence, found that IL-17A stimulation of alveolar epithelial cells increased the expression of α-SMA and TGF-β (27) (**Figure 2**). These findings are corroborated by data from animal models (18) and human biopsies (108).

NF-κB, a major transcriptional regulator of inflammation, acts downstream of IL-1β and IL-17A (109). NF-κB promotes fibrosis through indirect activation of TGF-β and α-SMA expression in myofibroblasts (110, 111).

Emerging evidence underscores the involvement of the IL-17 cytokine family in driving the transition from inflammation to fibrosis in IPF (112). IL-17 isoforms, including IL-17A, IL-17B, IL-17C, IL-17D, IL-17E, and IL-17F (113–116), have been implicated in mediating acute and chronic inflammation through innate and adaptive immunity. IL-17A is a key driver of fibrosis (12, 18, 27), while IL-17B and IL-17E further exacerbate the fibrotic response by promoting the secretion of inflammatory mediators such as TNF-α, IL-6, and IL-1β. IL-17C has been shown to contribute to epithelial damage and neutrophil recruitment, while IL-17D and IL-17F also regulate immune responses linked to fibrosis (117–119). Mechanistically, IL-17 signaling occurs through IL-17 receptors, leading to the activation of NF-κB and MAPK (120–123), pathways that enhance fibroblast activation and ECM deposition. IL-17E, also known as IL-25, has been shown to promote epithelial-to-mesenchymal transition (EMT) and fibroblast activation, contributing to collagen deposition and fibrosis progression (124). Additionally, IL-17B has been implicated in neutrophil recruitment and Th17 cell differentiation (125), which may further amplify inflammatory and fibrotic pathways. Targeting IL-17 pathways with specific inhibitors or monoclonal antibodies may represent a promising therapeutic strategy for IPF, as demonstrated by emerging preclinical studies (112).

In addition to well-characterized profibrotic cytokines, recent evidence suggests that IL-11 plays a significant role in the pathogenesis of IPF. Recent studies have identified IL-11 as a central effector of fibrosis, particularly affecting fibroblasts and AT2 cells. Ng et al., 2019 (126) demonstrated that IL-11 is upregulated in fibrotic lungs driving fibroblast activation, activated myofibroblast differentiation, and excessive extracellular matrix production. Importantly, genetic or pharmacological inhibition of IL-11 signaling was sufficient to attenuate fibrosis in multiple preclinical models, underscoring its potential as a therapeutic target. More recently, the study by Ng et al., 2024 (127) further expanded IL-11’s pathological role by linking it to AT2 cell dysfunction, showing that IL-11 impairs epithelial cell repair and contributes to epithelial-mesenchymal crosstalk that perpetuates fibrotic remodeling. Together, these findings position IL-11 as a profibrotic cytokine, affecting both mesenchymal and epithelial cells.

While many interleukins promote fibrogenesis, IL-22 has been identified as a cytokine with potential antifibrotic properties. IL-22, produced by immune cells such as T-helper 22 cells, primarily targets epithelial cells and has been implicated in tissue regeneration and protection. In the context of liver fibrosis, IL-22 has been shown to ameliorate fibrogenesis by inducing senescence in hepatic stellate cells (HSCs), thereby reducing ECM production (128). Moreover, treatment with IL-22 accelerated the resolution of liver fibrosis in mice (129). The hepatoprotective and antifibrotic functions of IL-22 suggest its therapeutic potential for the treatment of alcoholic liver disease (130). In the context of pulmonary fibrosis, emerging evidence also points to a protective role for IL-22, particularly through its effects on epithelial cells. Although IL-22 levels in the bronchoalveolar lavage fluid of IPF patients were comparable to those of healthy controls (131), Liang et al., 2013 (132) demonstrated that IL-22 mitigates epithelial-to-mesenchymal transition (EMT) in a bleomycin-induced model of pulmonary fibrosis. Furthermore, neutralizing IL-22 in this model resulted in increased extracellular matrix deposition and worsened fibrotic progression, suggesting that IL-22 plays a crucial role in limiting fibrogenesis in the lung. Further research is needed to elucidate the specific pathways through which IL-22 influences lung fibrosis and to assess its therapeutic potential in this context.

The inflammatory cytokines discussed here are produced not only by epithelial cells and fibroblasts but also by immune cells, which play a crucial role in IPF pathogenesis. Immune cells are present in the lung microenvironment during fibrosis and actively regulate fibroblast activity. Studies have shown that immune cells influence fibroblast proliferation, extracellular matrix deposition, and overall fibrotic remodeling, highlighting their significant contribution to fibrogenesis, which will be further explored.

### Immune cell contributions to fibrosis and associated mechanisms

Macrophages and monocytes are central to fibrosis progression. In IPF, circulating monocytes display a primed type I IFN phenotype, characterized by increased CD64 (FcγRI) expression, which correlates with fibrosis severity (133). Monocyte-derived macrophages, rather than resident alveolar macrophages, are implicated in fibrotic lung remodeling, exhibiting pro-fibrotic properties by secreting TGF-β and IL-13 (134, 135). Transitional macrophages in IPF lungs show elevated CD64 and CCL-2 expression, suggesting an inflammatory and fibrotic role (133). Additionally, increased monocyte recruitment is mediated by chemokines such as CCL-2, IL-6, and CSF-1 (136, 137), which drive their differentiation into pro-fibrotic macrophages (138). TGF-β plays a crucial role in macrophage-driven fibrosis. Once recruited to fibrotic tissues, monocyte-derived macrophages activate the Smad-dependent and Smad-independent TGF-β pathways, leading to fibroblast activation and ECM deposition (139). A recent study (140) has shown that lung macrophages are not essential during the initial inflammatory phase of bleomycin-induced lung injury but become key players during the later progressive fibrotic stage. Through macrophage depletion approaches and the use of a *Tgf-beta* (TGF-β) overexpression model, a specific subset of alternatively activated macrophages (marked by CD163, Ym1, and arginase-1) was found to significantly contribute to fibrosis by releasing pro-fibrotic mediators such as TGF-β, CCL18, and IGF-1. These factors support fibroblast persistence and extracellular matrix production. While Ly6C^hi^ monocytes do not appear to directly transform into these macrophages, they may play an indirect role in shaping their development or activity (140).

Recent reviews have highlighted the heterogeneity of macrophage phenotypes and their distinct roles in the progression and resolution of fibrosis (141–143). Beyond the classical M1/M2 paradigm, more refined subsets have been identified with specific fibrotic functions. For instance, various M2 subtypes—including M2a, M2b, M2c, and M2d—have been shown to play diverse roles in tissue repair, immune regulation, and fibrosis, primarily through the production of cytokines such as TGF-β and IL-10 (141–143). Additionally, macrophages expressing Sphingosine-1-phosphate receptor 2 (S1PR2) have been implicated in promoting pulmonary fibrosis via STAT6-dependent M2 polarization, and targeting this pathway has been shown to mitigate fibrotic responses (144). Matrix metalloproteinase-28 (MMP-28) has also been identified as a regulator of macrophage polarization, with its deficiency limiting M2 polarization and conferring protection against bleomycin-induced pulmonary fibrosis (145). Moreover, recent findings suggest that a subset of macrophages can undergo macrophage-to-myofibroblast transition (MMT), directly contributing to the myofibroblast pool in renal and lung fibrosis (146, 147).

Little is known for the role of Natural killer (NK) cells in IPF and most evidence derive from liver fibrosis. NK cells, known for their roles in antiviral and antitumor immunity, also significantly influence tissue inflammation and regeneration, thereby impacting fibrotic processes. For instance, interactions between NK cells and hepatic stellate cells (HSCs) can result in either the promotion or attenuation of fibrosis, depending on the specific microenvironmental context (148). Additionally, in advanced liver fibrosis, activated HSCs produce TGF-β, which can suppress NK cell antifibrotic functions, such as degranulation and interferon-gamma (IFN-γ) production, thereby promoting the progression of liver fibrosis (149).

Neutrophils exacerbate fibrosis through ROS and neutrophil extracellular traps (NETs). In IPF, increased neutrophil infiltration correlates with disease severity, with NETs releasing pro-fibrotic matrix metalloproteinases (MMPs). NETs also function as a scaffold for fibroblast migration and ECM deposition, further accelerating fibrosis. The interaction between neutrophils and fibroblasts is mediated by NETs, which release proteases such as MMP-9, contributing to fibroblast activation and tissue remodeling. Additionally, NET-associated damage-associated molecular patterns (DAMPs) activate TLR4 and RAGE signaling, perpetuating fibrotic responses (150, 151).

Regulatory T cells (T-reg), a subset of T-lymphocytes, migrate to the lungs and exhibit profibrotic properties by stimulating fibroblast proliferation through TGF-β, initiating fibrogenesis even in healthy mice (152, 153). In human studies, T-cells are found in the bronchoalveolar fluid of IPF patients (154), and the ratio of CD4+/CD8+ T-cells in the lungs correlates with disease prognosis (155). T-regs differentiate from CD4+ T-cells into Th17 cells under the influence of TGF-β, producing cytokines such as IL-17. The IL-17/IL-23 axis is crucial in linking T cell responses to fibrosis progression. IL-17A promotes fibroblast activation and ECM accumulation by engaging the NF-κB and p38 MAPK pathways, which synergize with TGF-β signaling to sustain myofibroblast activation (27). IL-23 supports Th17 differentiation (156), reinforcing fibrotic signaling and amplifying IL-17-mediated fibroblast proliferation (157).

Innate lymphoid cells (ILCs) are a recently identified class of immune cells that lack antigen-specific receptors, with the ILC2 subset playing a critical role in fibrosis. In IPF, elevated ILC2 populations secrete pro-fibrotic cytokines, including IL-4, IL-5, IL-9, and IL-13, which contribute to fibroblast activation and extracellular matrix deposition. IL-25, which is increased in IPF lungs, induces IL-13-expressing ILC2 expansion, while IL-33 further enhances ILC2 activation and M2 macrophage polarization, leading to increased IL-13 and TGF-β production (158–160).

B lymphocytes (B cells) contribute to fibrosis through antibody production and secretion of TNF-α and IL-6, which enhance fibroblast proliferation. Elevated BAFF (B cell activating factor) levels have been associated with fibrotic disease progression. B cell depletion therapies have shown promise in reducing fibrosis severity in experimental models, highlighting their pathogenic role in bleomycin induced IPF and scleroderma model (161, 162). IL-6, a key cytokine produced by B cells, acts through the JAK-STAT3 signaling pathway, which promotes TGF-β signaling (163) and lead to fibroblast differentiation into myofibroblasts and enhances ECM production (3, 163).

Immune cells play a fundamental role in fibrosis through complex interactions with fibroblasts and inflammatory mediators. By linking immune cell activation with molecular pathways such as TGF-β, IL-17/IL-23 and type I IFN this section underscores potential areas for therapeutic intervention. Future research should focus on modulating immune cell activity to mitigate fibrosis and improve clinical outcomes.

### Immune cell contributions to fibrosis resolution

Macrophages are central to fibrosis resolution due to their plasticity and ability to switch between pro-inflammatory (M1) and anti-inflammatory (M2) phenotypes (164). Upon exposure to anti-inflammatory cytokines like IL-4 and IL-13 or pro-reparative signals such as peroxisome PPAR-γ, macrophages polarize toward a reparative phenotype (165–167). Interestingly, macrophages are also important regulators of lipid metabolism in the lung and may play an important role in cell-cell communication between lipofiboblasts and other cell types. More specifically macrophages with active fatty acid oxidation and balanced cholesterol handling tend to adopt a pro-resolving (M2-like) phenotype, secreting cytokines like IL-4 and PPAR-γ agonists (168, 169). From the perspective of fibroblasts, this change favors a pro-resolving phenotype (25, 28).

Interestingly, the role of IL-10 in inflammation is likely context-dependent. IL-10 has been described as inflammatory under SARS-CoV-2 infection (55) but also anti-inflammatory in the context of metabolic reprogramming of macrophages (167).

These macrophages secrete matrix metalloproteinases (MMPs) which aid in the remodeling of the ECM and the resolution of fibrosis (170). Notably, depletion of macrophages during this recovery phase impairs ECM degradation and delays fibrosis resolution (140). Although the specific macrophage subtype responsible for ECM breakdown remains uncertain, M1 macrophages are strong candidates, as they produce MMPs such as MMP7 and MMP9. Elevated MMP9 levels have been observed in IPF lungs, potentially reflecting a failed reparative response due to concurrent increases in its inhibitor, TIMP-1 (171–173).

As aforementioned, a little is known for the role of NK cells in IPF. NK cells are traditionally known for their cytotoxicity, particularly in eliminating virally infected or malignant cells. However, in the context of fibrosis resolution, NK cells play a crucial role by targeting activated fibroblasts, including hepatic stellate cells HSCs, which are key drivers of liver fibrosis. NK cells exert cytotoxicity through the release of perforin and granzymes, inducing apoptosis in these fibroblasts. This process is crucial for preventing the excessive accumulation of ECM components and facilitating tissue repair (174). Furthermore, NK cells secrete cytokines like interferon-gamma (IFN-γ), which modulate the fibrotic process by altering the balance of pro-inflammatory and anti-inflammatory mediators (175, 176). IFN-γ has been shown to downregulate the activity of HSCs and promote the resolution of fibrosis by enhancing the phagocytosis of apoptotic cells and modulating TGF-β signaling (149). NK cells can also influence macrophage polarization, promoting a reparative macrophage phenotype that further supports tissue remodeling (177).

Regulatory T cells (Tregs) are essential for maintaining immune homeostasis and preventing excessive fibrotic responses. They suppress effector T cell activity and inhibit pro-inflammatory pathways that could exacerbate fibrosis (178). Tregs produce anti-inflammatory cytokines such as interleukin-10 (IL-10) (179, 180) which limit fibroblast activation and collagen deposition (181). In the context of fibrosis resolution, Tregs help balance immune tolerance with reparative responses. Tregs influence the ECM remodeling process by limiting the recruitment of fibroblasts through cytokines alleviating fibrinogenesis (182–184).

Dendritic cells (DCs) play a pivotal role in bridging innate and adaptive immune responses, especially in the context of fibrosis. They can adopt a tolerogenic phenotype, crucial for promoting fibrosis resolution and preventing chronic activation of fibrogenic pathways (185). Tolerogenic DCs facilitate regulatory T cell (Treg) differentiation by secreting IL-10, supporting immune tolerance and tissue repair (186). Moreover, DCs aid in the removal of apoptotic cells (187), preventing persistent inflammation (188). The interaction between DCs and Tregs is essential for balancing immune suppression and fibrosis resolution. DCs in fibrotic tissues may influence fibroblast behavior by secreting anti-inflammatory cytokines that limit ECM deposition and promote tissue repair (189).

Eosinophils are commonly associated with allergic responses but have also been implicated in the modulation of fibrosis. In certain contexts, eosinophils can secrete cytokines and growth factors, such as IL-4, IL-5, and TGF-β, which influence the activation of fibroblasts and the remodeling of the ECM. The role of eosinophils in fibrosis is complex, as they can have both pro-fibrotic and anti-fibrotic effects depending on the tissue microenvironment (190, 191). In some models of fibrosis, eosinophils contribute to ECM degradation by producing matrix metalloproteinases (MMPs), promoting tissue repair. However, in other contexts, eosinophils may exacerbate fibrosis by producing profibrotic cytokines that enhance the activation of fibroblasts and the deposition of collagen (192).

**Figure 3:**
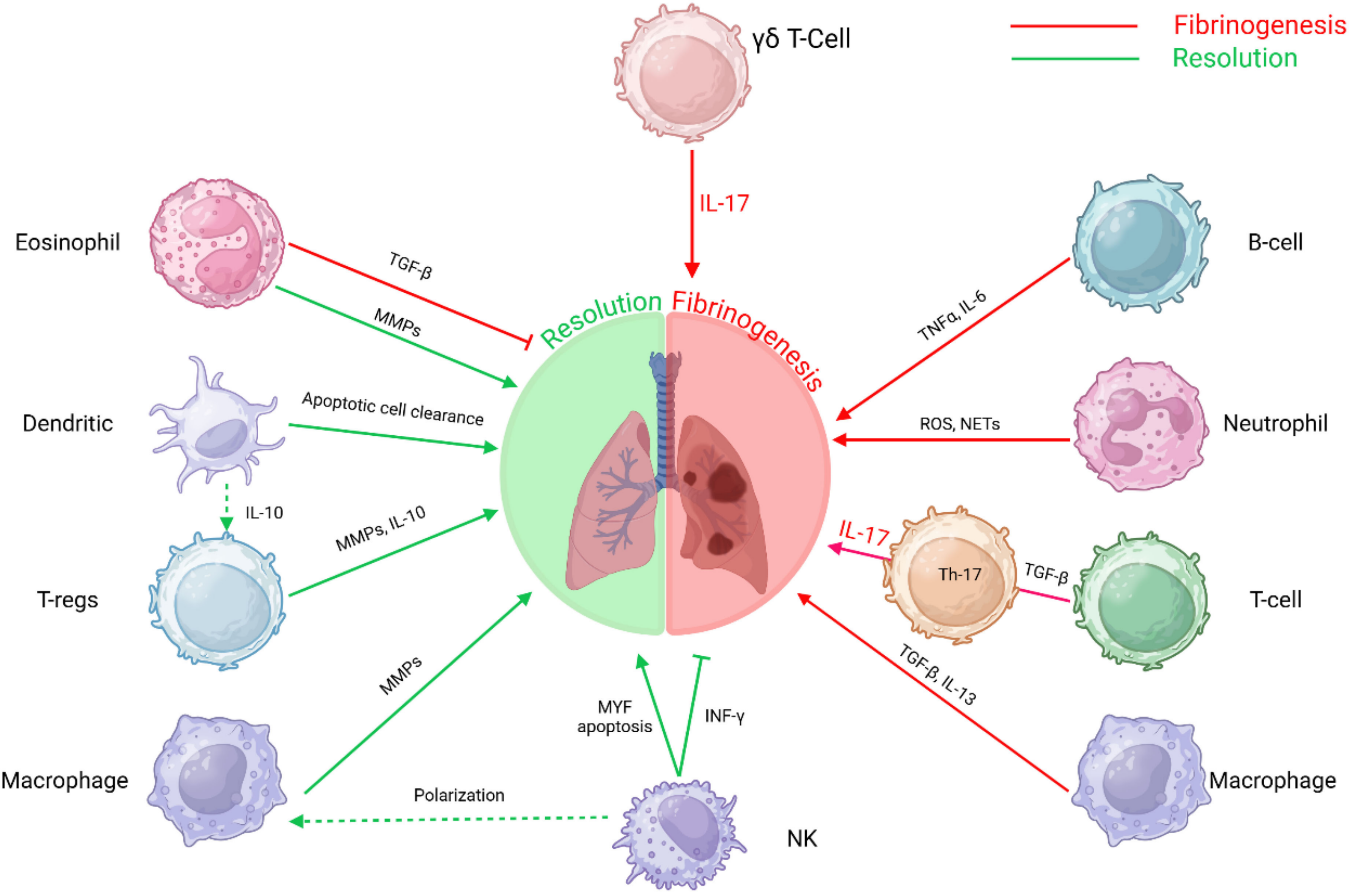
Immune cell interactions in fibrosis: This schematic depicts the intricate roles of various immune cells in the progression and resolution of fibrosis. The interplay between these immune cells and molecular pathways underscores the multifaceted nature of fibrosis, involving immune regulation, tissue remodeling, and inflammatory responses. T-helper 17 T-lymphocytes Th17 T-cells, B-lymphocytes B-cells, Gamma delta T-lymphocytes γδ T-Cells, T-regulatory cells (T-regs) and Natural Killer cells (NK).

The resolution of fibrosis is a multifaceted process involving the coordinated actions of various immune cells. Macrophages, NK cells, Tregs, DCs, and eosinophils each contribute uniquely to modulating fibrotic responses and promoting tissue repair (**Figure 3**). Understanding the intricate interactions among these immune cells offers potential therapeutic avenues for enhancing fibrosis resolution and restoring organ function.

## Conclusion

Fibrosis is a complex and progressive pathological process characterized by the excessive deposition of extracellular matrix (ECM) components, leading to tissue scarring and organ dysfunction. In idiopathic pulmonary fibrosis (IPF), chronic epithelial injury triggered by persistent oxidative stress, inflammation, viral infections, aging and metabolic dysregulation drives the activation of fibroblasts and their differentiation into myofibroblasts, which are responsible for ECM deposition and tissue stiffening. However, emerging research highlights the potential for fibrosis resolution through the reversible transition of myofibroblasts back to a lipofibroblast phenotype, which is associated with tissue repair and homeostasis. Lipofibroblasts, characterized by their lipid storage capabilities and close association with alveolar epithelial cells, play a crucial role in regulating alveolar niche homeostasis. Understanding the dynamic interplay between fibrotic and reparative cell states, as well as the molecular mechanisms driving fibrosis progression and resolution, offers promising therapeutic interventions for reversing fibrosis and restoring tissue function in IPF.

Inflammation plays a pivotal role in the pathogenesis of IPF, acting as both a driver and a perpetuator of fibrotic processes. Chronic inflammation, triggered by factors such as viral infections, oxidative stress, and metabolic dysregulation, leads to the persistent activation of pro-inflammatory cytokines, including IL-1β, IL-17A, and TGF-β. These cytokines create a self-sustaining loop that exacerbates tissue injury and fibrosis. For instance, IL-17A not only promotes fibroblast but also enhances TGF-β stability, further amplifying fibrotic responses. Additionally, oxidative stress, through the generation of ROS, activates key inflammatory regulators such as NF-κB, which in turn upregulates pro-inflammatory genes. This inflammatory setting disrupts normal tissue repair mechanisms, leading to the excessive deposition of ECM components and the progression of fibrosis. Understanding the intricate interplay between inflammation and fibrosis is crucial for developing targeted therapies that can break this vicious cycle and promote tissue repair.

The reversible transition between activated myofibroblasts and lipofibroblasts represents a critical mechanism in the resolution of pulmonary fibrosis. Activated myofibroblasts, characterized by their role in ECM deposition and tissue contraction, are central to fibrotic progression. However, studies demonstrated that activated myofibroblasts can revert to a lipofibroblast phenotype, which is associated with tissue repair and homeostasis. Lipofibroblasts, marked by their lipid storage capabilities and close association with alveolar epithelial cells, play a key role in regulating inflammation and promoting ECM reabsorption. This phenotypic switch is regulated by metabolic and signaling pathways, including PPAR-γ and TGF-β. Although metformin has shown promising antifibrotic effects in preclinical models, *post hoc* analyses in IPF patients have not consistently demonstrated a clear therapeutic benefit (193). In contrast, a recent cohort study involving IPF patients with type 2 diabetes mellitus reported that metformin use was associated with a significant reduction in all-cause mortality and hospitalization rates (194). These contrasting findings emphasize the need for well-designed, prospective clinical trials to rigorously evaluate the efficacy of metformin in IPF. The plasticity of fibroblasts highlights the potential for therapeutic interventions that target this reversible switch, offering a novel approach to fibrosis resolution by restoring the balance between fibrotic and reparative cell populations.

Recent evidence necessitates a re-evaluation of the notion that IPF is not driven by chronic inflammation. Increasing findings highlight inflammation as a key factor in fibrosis progression (12, 18, 52). In the study of Tsukui et al., 2024 (18), the authors proposed a novel intermediate inflammatory fibroblast derived from *Scube2* ^+^ alveolar fibroblast population which will be discussed here. The authors provide evidence that, at day 6 in a bleomycin-induced murine model, genetic ablation of alveolar fibroblasts led to increased γδ T cell infiltration and upregulated IL-17A expression, indicating an inflammatory response. On the other hand, even in WT mice, γδ T cells and neutrophils were present, underscoring an immune component in fibrosis, independent on fibroblast presence. This is also supported by the finding that, IL-17A neutralization mitigated weight loss and mortality in alveolar fibroblast-ablated mice. Fibroblasts express IL-17RA (107) and respond to IL-17A signaling (195). Given the lung’s complex cellular environment, investigating IL-17A neutralization in WT mice could clarify whether its effects are immune cell-dependent, fibroblast-mediated, or both. Furthermore, the authors to support the ‘intermediate’ inflammatory fibroblast theory found that inflammatory fibroblast markers peak mid-pseudotime before declining, as fibrotic fibroblast markers increase. This aligns with the dual role of TGF-β, which exhibits both immunomodulatory and pro-fibrotic properties. TGF-β primarily exerts immunosuppressive effects by inhibiting the activation of T cells, macrophages, and dendritic cells while promoting the expansion of regulatory T cells (Tregs), which help maintain immune tolerance and suppress excessive inflammatory responses (196). Additionally, TGF-β downregulates pro-inflammatory cytokines such as interleukin-1 (IL-1), and interleukin-6 (IL-6), thereby contributing to the resolution of inflammation (197). However, in chronic inflammatory conditions and fibrosis, TGF-β can drive pathogenic responses by promoting extracellular matrix deposition and immune cell infiltration, leading to tissue scarring and dysfunction (198). In the study of Tsukui et al., 2024 (18) inflammatory fibroblast frequency fluctuated minimally during fibrosis progression, while alveolar and fibrotic fibroblast populations altered. Localization assays revealed inflammatory fibroblasts adjacent to, but not overlapping, fibroblastic foci, suggesting a role in fibrosis formation. This persistent presence of inflammatory fibroblasts points towards symbiotic interaction than intermediate population. Lineage tracing upon immune checkpoint inhibition or reprogramming of myofibroblast towards lipofibroblast phenotype could determine the transdifferentiation into intermediate inflammatory fibroblasts potential.

To support the ‘intermediate’ inflammatory fibroblast theory, the authors utilize a Transforming growth factor beta receptor 2 conditional knock-out (*Tgfbr2*-cKO) model in alveolar fibroblasts, demonstrating that fibrosis is prevented despite excessive inflammation. Moreover, the observed increase in inflammatory markers and decrease in fibrotic markers in lineage-labeled alveolar fibroblasts suggest that TGF-β signaling is essential for driving inflammatory fibroblasts toward a fibrotic phenotype.

**Figure 4:**
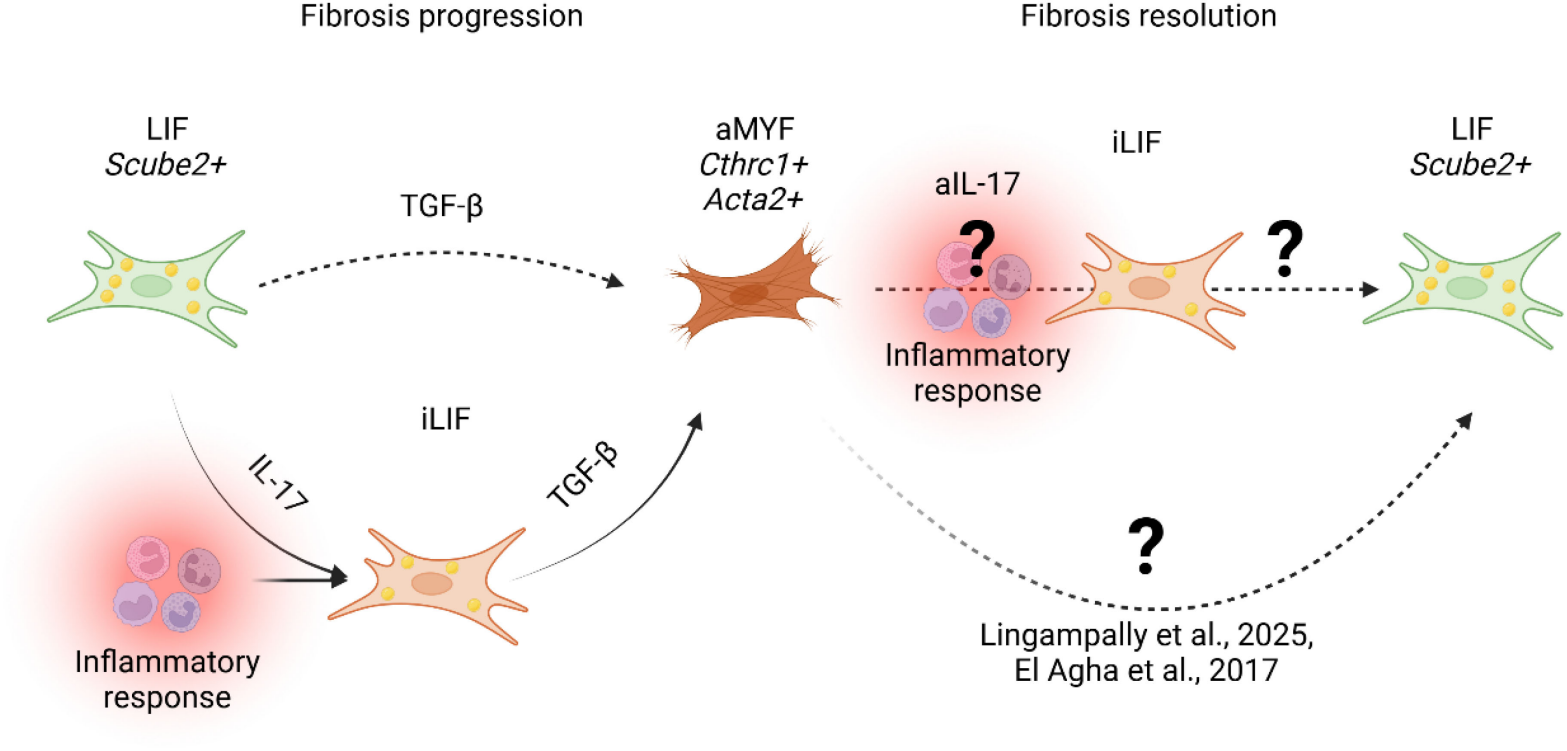
Proposed model of fibrosis progression and resolution. During fibrosis progression, lung alveolar lipofibroblasts (LIF *Scube2^+^*) transition into an inflammatory LIF-expressing state (iLIF) under IL-17-mediated inflammation (Tsukui et al., 2024). iLIF, driven by TGF-β, differentiate into activated myofibroblasts (aMYF, *Cthrc1^+^ Acta2^+^*), promoting fibrosis. Resolution involves myofibroblast reversion to lipofibroblasts (LIF) (25, 26, 52). Tsukui et al., 2024 suggest IL-17 inhibition (aIL-17) modulates iLIF, yet mechanisms governing iLIF reversion to LIF remain unclear.

It is proven that conditional *Tgfbr2* knockout sensitizes cells to IL-17A-induced chemokine secretion (199) and induces inflammation (200). Thus, all these information point towards further research on the intermediate inflammatory fibroblast theory, as indications may point towards fibroblasts responsive to inflammation enhancing the inflammatory alveolar niche (**Figure 4**).

Pharmacological interventions targeting inflammation, oxidative stress, and fibroblast plasticity are significantly promising for the treatment of IPF. Anti-inflammatory agents, such as IL-17 inhibitors, have shown potential in reducing fibrotic responses by disrupting the IL-1β–IL-17A–TGF-β axis. Antioxidants, including ALDH2 inducers, mitigate oxidative stress and its downstream effects on fibrosis by reducing ROS production and TGF-β activation. Additionally, metabolic modulators like PPAR-β/δ agonists would be of interest to study the ability to promote the reversion of myofibroblasts to lipofibroblasts, thereby facilitating fibrosis resolution. These interventions not only target the underlying mechanisms driving fibrosis but also offer the potential to restore normal tissue architecture and function. Future research should focus on optimizing these therapeutic strategies, potentially through combination therapies, to enhance their efficacy and potentially improve outcomes for patients with IPF.
